# Studies of Ribonucleotide Reductase in Crucian Carp—An Oxygen Dependent Enzyme in an Anoxia Tolerant Vertebrate

**DOI:** 10.1371/journal.pone.0042784

**Published:** 2012-08-14

**Authors:** Guro K. Sandvik, Ane B. Tomter, Jonas Bergan, Giorgio Zoppellaro, Anne-Laure Barra, Åsmund K. Røhr, Matthias Kolberg, Stian Ellefsen, K. Kristoffer Andersson, Göran E. Nilsson

**Affiliations:** 1 Department of Molecular Biosciences, University of Oslo, Oslo, Norway; 2 Laboratoire National des Champs Magnétiques Intenses, LNCMI-G, UPR 3228, CNRS, Grenoble, France; 3 Lillehammer University College, Lillehammer, Norway; University of Wales Bangor, United Kingdom

## Abstract

The enzyme ribonucleotide reductase (RNR) catalyzes the conversion of ribonucleotides to deoxyribonucleotides, the precursors for DNA. RNR requires a thiyl radical to activate the substrate. In RNR of eukaryotes (class Ia RNR), this radical originates from a tyrosyl radical formed in reaction with oxygen (O_2_) and a ferrous di-iron center in RNR. The crucian carp (*Carassius carassius*) is one of very few vertebrates that can tolerate several months completely without oxygen (anoxia), a trait that enables this fish to survive under the ice in small ponds that become anoxic during the winter. Previous studies have found indications of cell division in this fish after 7 days of anoxia. This appears nearly impossible, as DNA synthesis requires the production of new deoxyribonucleotides and therefore active RNR. We have here characterized RNR in crucian carp, to search for adaptations to anoxia. We report the full-length sequences of two paralogs of each of the RNR subunits (R1i, R1ii, R2i, R2ii, p53R2i and p53R2ii), obtained by cloning and sequencing. The mRNA levels of these subunits were measured with quantitative PCR and were generally well maintained in hypoxia and anoxia in heart and brain. We also report maintained or increased mRNA levels of the cell division markers proliferating cell nuclear antigen (PCNA), brain derived neurotrophic factor (BDNF) and Ki67 in anoxic hearts and brains. Electron paramagnetic resonance (EPR) measurements on *in vitro* expressed crucian carp R2 and p53R2 proteins gave spectra similar to mammalian RNRs, including previously unpublished human and mouse p53R2 EPR spectra. However, the radicals in crucian carp RNR small subunits, especially in the p53R2ii subunit, were very stable at 0°C. A long half-life of the tyrosyl radical during wintertime anoxia could allow for continued cell division in crucian carp.

## Introduction

Crucian carp (*Carassius carassius*) has the extraordinary ability to live in the complete absence of oxygen (anoxia) for up to several months under the ice in small ponds [1], [2]. In part, this is made possible by its ability to produce ethanol as the end-product in glycolysis, thus avoiding lactate accumulation and acidosis [3]. By suppressing activity in anoxia to save ATP, it can survive throughout the winter solely on glycolytic ATP production from glycogen, which is primarily stored in the exceptionally large liver [4], [5].

However, in addition to ATP synthesis, there are numerous processes in the vertebrate body that are more or less dependent of molecular oxygen. So far, such processes have received little attention in anoxia tolerant vertebrates, even if it includes such basal functions as DNA synthesis, and therefore cell division. Ribonucleotide reductase (RNR) catalyzes the reduction of ribonucleotides to the corresponding deoxyribonucleotides (dNTPs), essential building blocks for DNA. All described RNR variants, ranging from virus to vertebrate RNRs, share a common reaction mechanism depending on a conserved cysteine that must be converted to a thiyl radical for catalysis [6]. However, the method for forming this radical differs between the RNRs [7], [8]. All vertebrates, and all eukaryotes together with some prokaryotes and viruses contain class I RNR and these are characterized by being dependent on oxygen for generating the radical, often a tyrosyl radical [7]. Class I is further divided into class a–c, where class Ia is the classical class I RNR found in all vertebrates and eukaryotes. Class II RNRs use deoxyadenosylcobalmin as cofactor instead of the R2 subunit and does not depend on oxygen for the generation of radical. This variant is found in different bacteria, and a few unicellular eukaryotes. Finally, with the help of an iron-sulphur protein and S-adenosyl methionine the class III RNR found in some bacteria forms a glycyl radical that is lost in the presence of oxygen [9].

Class Ia RNR of eukaryotes consists of the following subunits: the large subunit, R1, containing the active site, and the small subunit, R2 or p53R2, where a tyrosyl radical is formed in the reaction between oxygen and a ferrous di-iron cluster. When substrate and effectors are bound to the R1 subunit, the radical is transported from the tyrosyl radical in the small subunit, via a chain of conserved hydrogen bonded amino acids, to the cysteine at the active site in R1 [10]–[13]. The radical is not consumed in the reaction and after the dNTP has been formed, the radical is transported back along the same pathway for use in the next catalytic cycle [8], [14], [15]. However, the tyrosyl radical is readily lost and in mammals it has a half-life of about 10–60 min [16]–[18].

In preparation for mitosis, the dNTP concentration in the cell increases 20 fold [19]. The activity of RNR is restricted to S and G_2_ phase of the cell cycle, through S-phase specific expression of the R2 subunit [20], [21], and degradation of R2 in mitosis [22]. R1 mRNA expression is also mostly S-phase specific [21], but the R1 protein is more long-lived and its level is kept almost constant throughout cell cycle in proliferating cells [23]. A particular form of R2, called p53R2, is expressed at low levels in all stages of the cell cycle, and can also be induced by p53. This variant has been shown to be important for nucleotide production for DNA repair and mitochondrial DNA replication [19], [24]–[26].

Without oxygen, no regeneration of the tyrosyl radical in class Ia R2/p53R2 can occur, which should lead to a halt in dNTP synthesis, and therefore stop in cell division [27], [28]. Interestingly, measurements of bromodeoxyuridine (BrdU) incorporation have indicated that crucian carp maintain cell division in anoxia [29]. While bacterial class II and III RNR enzyme can generate the thiyl radical without oxygen [30], [31], at present knowledge, this is not possible for the class Ia enzymes. Still, it is possible that the ability of different vertebrate RNRs to maintain a functional radical may vary. One previous study of crucian carp RNR R2 has indicated a conserved sequence when compared to other vertebrates [29], but since crucian carp often has more variants of each gene than other fishes due to a genome duplication in the *Carassius* lineage [32]–[34], we have here attempted to identify all RNR variants in this species. Furthermore, we have measured their expression in normoxia, hypoxia and anoxia with quantitative PCR (qPCR), along with markers of cell division. In addition, we have *in vitro* expressed the crucian carp R2 and p53R2 variants and studied their radical environment with X-band and high field/high frequency (HF) electron paramagnetic resonance (EPR). Also their di-iron-oxygen cluster sites in the mixed-valent (Fe^II^Fe^III^) form were studied with X-band EPR. For comparison, we also performed EPR and HF-EPR measurements on mouse and human p53R2.

We here present the full-length sequence of two crucian carp RNR R1, R2 and p53R2, twice as many as is found in zebrafish and mammals. Furthermore, we show that all crucian carp RNR variants are very similar to their mammalian counterparts, both in nucleotide sequence and radical environment. We found that the radical in the crucian carp p53R2ii variant was very stable in anoxia, which may allow extended function during anoxic overwintering. We speculate that this can explain why crucian carp can do the “impossible”: having cell division in anoxia.

## Materials and Methods

All chemicals were purchased from Sigma, unless otherwise is stated.

### Animals

Crucian carps of mixed sex were caught in Tjernsrud pond near Oslo. They were kept at the aquarium facility of the Department of Molecular Biosciences, University of Oslo, in tanks (100 fish/500 l) continuously supplied with aerated and dechlorinated Oslo tap water (17°C). The fish were fed daily with commercial carp food and were acclimated to the experimental temperature for at least four weeks. Fish were not fed during hypoxia or anoxia exposure. The experiments were approved according to Norwegian animal research guidelines at an approved animal facility (Norwegian Animal Research Authority approval nr 155/2008).

### Cloning of crucian carp RNR subunits and markers of cell division

One RNR R2 variant has been previously cloned in crucian carp [29]. We designed fragment primers in the Primer3 program [35] based on RNR R1, R2, p53R2, brain-derived neurotrophic factor (BDNF), proliferating cell nuclear antigen (PCNA) and Ki67 sequences from other vertebrates found on NCBI and Ensembl (Table 1). The cloning was performed as previously described [36]. In short, the cloning primers (Invitrogen) and cDNA made from brain, heart, liver and intestine (following the procedure described under qPCR assay), were used in a PCR with Platinum® *Taq* DNA Polymerase (Invitrogen). PCRs with heavily degenerated primers designed based on yeast RNR sequences were also performed, but no new RNR sequences were obtained from these attempts. The PCR products were ligated into pGEM®-T Easy Vector System I (Promega), following the protocol of the manufacturer. The plasmids were subsequently used to transform competent *E. coli* bacteria. Positive colonies were used in an additional PCR with M13F2 and M13R primers (Invitrogen), and PCR products with the right size were cleaned with ExoSAP-IT (VWR) and sequenced with M13 primer at the sequencing service at Department of Biology, University of Oslo.

**Table 1:** Primers used for cloning, RACE, qPCR and in vitro expression.

**Cloning primers (fragment)**	
Sequence obtained	Primer sequence (5′ - 3′)
RNR R1i	F: ATTGCGGCTGCWATTGAAAC
	R: TTGCAAACTGCAACCTCATC
RNR R1ii	F: TGGAGCGTTCATATYTGYTG
	R: TTGCAAACTGCAACCTCATC
RNR R2i	F: TTCCCTGAAAGACGANGAGA
	R: TTCATGCCAATGAGCTTHAC
RNR R2ii	F: CGCTTTGTCATTTTCCCMAT
	R: AGTCGGTCAGCCACAAACTC
RNR p53R2i	F: GCACAGGCTTCTTTCTGGAC
	R: AGTGTAGGCCCTCRTCYCTG
RNR p53R2ii	F: CTTTCTTTGCGGCAAGTGAT
	R: AGTGTAGGCCCTCRTCYCTG
BDNF	F: ATCAGCAACCAAGTGCCTTT
	R: GCCCCTCTTAATGGTCAATG
PCNA	F: GGAGGCTCTGAAAGACCTGA
	R: GCACTGGCTCATTCATCTCA
Ki67	F: CGCAGGTATCGAAAGAGCAT
	R: GAGGTGGGTACTCAAACCTGA
**RACE primers**	
Sequence obtained	
RNR R1i	5′: AGAGCTGTGGCCGGTTGG
	3′: GCATGCCATCATTGAGTCGCAGA
RNR R1ii	5′: AGAGCTGTGGCCGGTTGG
	3′: CAGCAGAACCTGGGCACCATCA
RNR R2i	5′: GCAACGGGCTTCAGTCACTTGGA
	3′: TCATGCCTGGACTCACCTTCTCCA
RNR R2ii	5′: TCCATGGCAATCTGGAAACCGTA
	3′: GCCCGTTGCTTCTACGGTTTCCA
RNR p53R2i	5′: GGCCCAGTCTGCTTTTCGCCTTA
	3′: CCATCTGCTGACCGAGTGAAAGACA
RNR p53R2ii	5′: GGCCCAGTCTGCTTTTCGCCTTA
	3′: GGTCCAGAGGTTCAGTCAGG
**qPCR primers**	
Sequence amplified	
RNR R1i (E_B_ = 1.72; E_H_ = 1.83)	F: GAGTACACCAGTAAGGATGAGGTG
	R: TCTTCACAATGACTTTTGTAACAGAG
RNR R1ii (E_B_ = 1.79; E_H_ = 1.86)	F: CAGAGAGCTAGAGATCTGTTTTACG
	R: TCACCACACGCTTAGCTCTT
RNR R2i (E_B_ = 1.86; E_H_ = 1.89)	F: AAACCTTCGGAGGCAACC
	R: CCAGCTCCAGCAGAAGTCTA
RNR R2ii (E_B_ = 1.87; E_H_ = 1.90)	F: GCTGGGATTTGACAAGGTTT
	R: GCTGGTACTCGCCAACTCTCT
RNR p53R2i (E_B_ = 1.84; E_H_ = 1.90)	F: CTAGGATTGCCCAAAGTTTTC
	R: TCCAAGTCGTTGGTATTCACC
RNR p53R2ii (E_B_ = 1.88; E_H_ = 1.90)	F: AAGAGGTGCAGATCCCAGAG
	R: GCATGGTGTGAATTGCATTG
PCNA (E_B_ = 1.83; E_H_ = 1.89)	F: CTTTGGCACTTGTCTTTGAAAC
	R: TTCACCACACAGCTGTATTCCT
BDNF (E_B_ = 1.87)	F: AGGTCCCCGTGACTAATGGT
	R: AGCCGATCTTCCTTTTGCTA
Ki67 (E = _B_1.78; E_H_ = 1.83)	F: GAGCTAAATGAGAACAAGGAGCTA
	R: CCATGCTTTAAACATTCCGACT
mw2060 (E_B_ = 1.82; E_H_ = 1.89)	F: CTGACCATCCGAGCGATAAT
	R: AGCAAGCTGTTCGGGTAAAA
**CDS primers with restriction enzyme sites** *	
Sequence amplified	
RNR R2i	F: GGAATTCCAT**ATG**CAGTCAACTCGCTCT ^1^
	R: AGGGTCGA**CTA**AAAATCAGCATCCAGC ^2^
RNR R2ii	F: GGAATTCCAT**ATG**TCGTCAACTCGCTCTC ^1^
	R: AGGGTCGA**C** **TA**AAAATCAGCATCCAGTCTG ^2^
RNR p53R2i	F: GGAATTCCAT**ATG**GAATATCAGAACGGTCACAG ^1^
	R: AGGAAGC**TT** **A**GAAATCTGCATCGAGAGTGAA ^3^
RNR p53R2ii	F: GGAATTCCAT**ATG**AACTCCAGCACAAGCA ^1^
	R: AGGAAGC**TT** **A**GAAATCTGCATCAAGAGTGAATT ^3^

Mean qPCR primer pair efficiencies (E) are given in brain (B) and hearts (H).

* Start and terminal codons are highlighted in bold. Restriction sites (^1^NdeI, ^2^SalI, ^3^HindIII) are underlined.

To obtain full-length sequences of RNR variants, gene-specific primers were designed for each variant (Table 1) and used in RACE-PCR (rapid amplification of cDNA ends PCR) with cDNA library made with SMART RACE cDNA Amplification Kit (Clontech). The mRNA used in the kit was extracted from brain, liver, heart, intestine and gill total RNA using Dynabeads® (Invitrogen). The amplified 3′ and 5′ ends were sequenced as described above and aligned with the fragment sequences. Homology modelling of the structure of crucian carp RNR R1, R2 and p53R2 variants were performed using the SWISS-MODEL server [37], based on the structures of mammalian RNR subunits (PDB identity: 3HNC (human R1), 1W68 (mouse R2) and 3HF1 (human p53R2), respectively). Phylogenetic trees were made with PHYLIP Phylogeny Inference Package (Protdist and Neighbor-joining, Version 3.67; http://evolution.genetics.washington.edu/phylip.html), based on the cloned crucian carp sequences aligned with RNR sequences from several fish and tetrapod species found at GenBank.

### Anoxia exposure and tissue sampling

Crucian carps (30.9±2.3 g; n = 40; 9.3–10.5°C) were exposed to different oxygen levels in the following six treatments: normoxia, 7 days of hypoxia, 1, 3 or 5 days of anoxia (post hypoxia), and 5 days reoxygenation (post 5 days of anoxia) in separate, dark tanks (minimum 1 l/fish).

Anoxic conditions were obtained by running the water through a narrow, 2 m high column bubbled with N_2_ gas (AGA) before letting it into the experimental tanks (flow rate 4 l h^−1^). N_2_ gas was also bubbled in the tanks. Oxygen content of the inflowing water was continuously monitored with a galvanic oxygen electrode, Oxi 340i (WTW), and was kept below the detection limit for the electrode, which was 0.01 mg l^−1^ (pO_2_∼0.13 mmHg).

Hypoxic conditions were obtained the same way, but oxygen content of inflowing water was kept at 0.6–0.9 mg O_2_ l^−1^ (pO_2_∼9–12 mmHg), resulting in 0.2–0.3 mg O_2_ l^−1^ (pO_2_∼3–5 mmHg) in the outflowing water due to oxygen consumption of the fish (flow rate ∼8 l h^−1^). Normoxic controls were kept in an identical tank, but with air bubbling (pO_2_∼157 mmHg).

At the time of sampling, 8 to 10 fish were killed by a sharp blow to the head and subsequent cutting of the spinal cord. Heart (ventricle) and brain tissue were dissected out and tissues were immediately snap-frozen in liquid N_2_, and stored at −80°C until RNA extraction. Crucian carp can survive more than two weeks of anoxia at the present temperature [4] and no mortality was seen during these experiments.

### qPCR assay

qPCR primers were designed from the crucian carp RNR R1, R2 and p53R2 (two variants of each subunit), PCNA, Ki67 and BDNF sequences identified by cloning and sequencing, using Primer3 (Table 1). At least one primer in each pair was placed at an exon-exon boarder, or the two primers in each pair were separated by an intron. All primers used in the experiment gave one peak in the melting curve analysis, and amplified the desired cDNA, as verified by cloning and sequencing of the qPCR product according to the cloning procedure described above. Total RNA was extracted with Trizol (Invitrogen) following the manufacturer's protocol. The tissue was weighed, frozen and quickly added 15 µl Trizol per mg tissue. Extraction was done in a random order (n = 6–10). An external mRNA standard, mw2060 [38] was added to the same tube (100 pg mw2060 per mg tissue) prior to homogenization. Total RNA concentration was measured with NanoDrop 2000 UV-Vis Spectrophotometer (Thermo Fisher Scientific, DE, USA). 2 µg of total RNA was DNase treated with DNA-free™ (Ambion) and used in cDNA synthesis with oligo(dT)_18_ and SuperscriptIII (Invitrogen). qPCR reactions were performed with a LightCycler 480 Real-Time PCR System (Roche) with 3 µl 1∶10 cDNA and 0.5 µM of each primer, total 10 µl. All reactions were run in duplicate on different plates. The following qPCR program was used: 95°C for 10 min, 42 cycles of 95°C for 10 s, 60°C for 10 s, and 72°C for 10 s. Finally, a melting temperature assay was conducted to assess the specificity of the reaction, consisting of the following program: 95°C for 5 s and 65°C for 10 s, followed by a gradual increase in temperature to 97°C, where the melting temperature of the qPCR was measured. The efficiency for each qPCR reaction was calculated with the LinReg software [39], [40], and the mean of all efficiencies for each pair in each tissue was used in the calculations (given in Table 1). The Cp values were obtained by the LightCycler 480 software with the second derivative maximum method. The relative mRNA level was calculated with the following formula: (E_mw2060_∧Cp_mw2060_)/(E_tar_∧Cp_tar_), where E is the mean efficiency for the primer pair, Cp is the mean Cp value for the two duplicate qPCR reactions, and tar is the target gene. mw2060 is the external standard used to normalize all qPCR data, according to previously described procedures [38].

### Protein expression, purification and EPR experiments

Crucian carp RNR R2i, R2ii, p53R2i and p53R2ii proteins were *in vitro* expressed as previously described [41]. First, cDNA was made from a mix of brain, gill, intestine and liver as described above, except that 0.2 ng/ul random primers were included in the reaction mix. To amplify the coding domain sequence (CDS) of crucian carp RNR variants and include restriction enzyme sites at each side, a PCR was performed with this cDNA and primers binding to each side of the CDS, which included restriction enzyme sites (Table 1), using Phusion® Hot Start II High-Fidelity DNA Polymerase (Finnzymes). The PCR product was inserted into pJET1.2 cloning vector (Fermentas) and subsequently cut and ligated into pET22b plasmid (Novagen), which was transformed into BL21 (DE3) Gold *E. coli* bacteria (Stratagene). Bacteria were grown, harvested and proteins were purified as previously described, with slight modifications [41]. In short, the bacteria were amplified before RNR protein production was induced by addition of 1 mM isopropyl-β-d-thiogalactopyranoside (IPTG). Cells were harvested after 16 h and frozen in liquid nitrogen before lysed with X-press [42]. After removal of DNA followed by protein precipitation, the RNR protein was purified using Äkta™ Purifier System (GE Healthcare). The protein solution was desalted on a 2×5 ml HighTrap desalting column (GE Healthcare) before being applied on a 2×5 ml HiTrap Q anion exchange column (GE Healthcare) and eluted by a 0–400 mM KCl gradient in 50 mM TrisHCl (pH = 7.5). The final purification step was performed using a Sephadex G25 gel filtration column (GE Healthcare) with 50 mM TrisHCl or 50 mM HEPES with 100 mM KCl (pH = 7.5). Human and mouse p53 protein were obtained as previously described [43].

The samples used for X-band and HF-EPR measurements were prepared as previously described [44]. In short, crucian carp and mammalian RNR R2 or p53R2 proteins were treated with 7× Fe^II^ ((NH_4_)_2_Fe(SO_4_)_2_·6H_2_O) per dimer of R2/p53R2 for reconstitution of the di-iron-oxygen cluster and generation of the tyrosyl radical site. The protein concentration was ∼200 µM in each EPR sample (calculated using molar extinction coefficient ε of 124000 M^−1^ cm^−1^/dimer at 280 nm), except for X-band and HF-EPR R2i samples, which were ∼60–80 µM dimer and human p53R2 HF-EPR samples which was ∼600 µM dimer. Samples were incubated at 0°C for 10 minutes with O_2_ present (ambient air), transferred to EPR tubes and quick-frozen by immersion in liquid nitrogen.

Mixed-valent forms of R2 or p53R2 subunits were obtained by reducing the tyrosyl radical-containing sample (see above) upon addition of 1.65 mM phenazine methosulfate (electron transfer mediator) and 1.65 mM dithionite (reductant) to the reconstituted samples as previously described [45].

EPR spectra were recorded at X-band on a Bruker Elexys 560 EPR spectrometer fitted with a Bruker ER41116DM dual-mode cavity, using a He-flow cryostat (ESR 900, Oxford Instruments). All spectra were measured under non-saturating microwave power conditions. The tyrosyl radical content was estimated by comparing the double integrated signal intensity with a standard solution containing 1 mM Cu^II^ EDTA in 50 mM HEPES, pH 7.5, 20% (v/v) glycerol, recorded under identical experimental conditions (temperature, gain, microwave power). The spin concentration was about one tyrosyl radical per dimer for R2ii and p53R2i. In a different context, two of these spectra have been published in a recent review [46].

The HF-EPR measurements, giving the low-temperature 285 GHz spectra, were recorded on a 95 GHz Gunn oscillator (Radiometer Physics, Germany) coupled to a frequency tripler as the frequency source. A superconducting magnet with a maximum field of 12 T at 4.2 K (Cryogenics Consultants, UK) was also coupled to the oscillator for the main magnetic field. Temperature of the samples was controlled by an Oxford Instrument cryostat and the light transmitted through the sample was detected with a ‘hot electron’ InSb bolometer as previously described [47], [48].

The stability of the tyrosyl radical was assessed by incubating reconstituted samples for 24 h in an anoxic atmosphere at 0°C and subsequently 1 h in a normoxic atmosphere at room temperature. The samples were transferred to EPR tubes after each treatment, before EPR spectra were recorded at X-band (77 K) as described above. The signal was double integrated and compared to the signal from a control sample which was immediately frozen after reconstitution.

### Statistical analysis

Levels of mRNA are expressed as mean values ± s.e.m, and tested for significant differences with one-way ANOVA with Dunnett's post-test with the normoxic group as control group. Statistical analyses were performed using JMP8 computer software (SAS Institute Inc.).

## Results

### Full-length cloning of crucian carp RNR subunits

Two paralogs (genes related by duplication) of each RNR subunit were identified by cloning and sequencing, and their full-length mRNA sequences were obtained by RACE. GeneBank accession numbers are given in parenthesis.

The two RNR R1 paralogs, R1i (JQ679004) and R1ii (JQ679005) had coding domain sequences (CDS) that translated into products of 793 amino acid (aa) residues. They were both highly similar to the R1 found in mouse and zebrafish, see amino acid sequence alignment in Fig. S1. The amino acid sequence showed conservation of all the important functional sites, such as active sites residues (Cys218, Asn427, Cys429, Glu431 and Cys444), radical transport residues (Tyr737 and Tyr738) and thioredoxin interaction sites (Cys788 and Cys791). Similarity of crucian carp RNR amino acid sequences to those of mouse and zebrafish, and similarities between crucian carp paralogs, are summarized in Table 2.

**Table 2:** Homology of crucian carp RNR subunits (amino acid sequences).

	Paralogs in crucian carp	Zebrafish	Mouse
R1i	96%	93%	89%
R1ii		94%	89%
R2i	95%	96%	81%
R2ii		94%	81%
p53R2i	96%	94%	80%
p53R2ii		93%	80%

The two RNR R2 paralogs, R2i (JQ679006) and R2ii (JQ679007) had CDSs translating into 385 aa and 386 aa long products, respectively, and were also similar to their mammalian counterparts (see amino acid sequence alignment in Fig. S2). All amino acid residues involved in important functional sites, such as in radical site (Tyr172/173), radical transport (Trp98/99, Arg260/261, Asp261/262 and Tyr365/366) and iron ligands (Asp134/135, Glu165/166, His168/169, Glu228/229, Glu262/263 and His265/266) were conserved.

**Figure 1:**
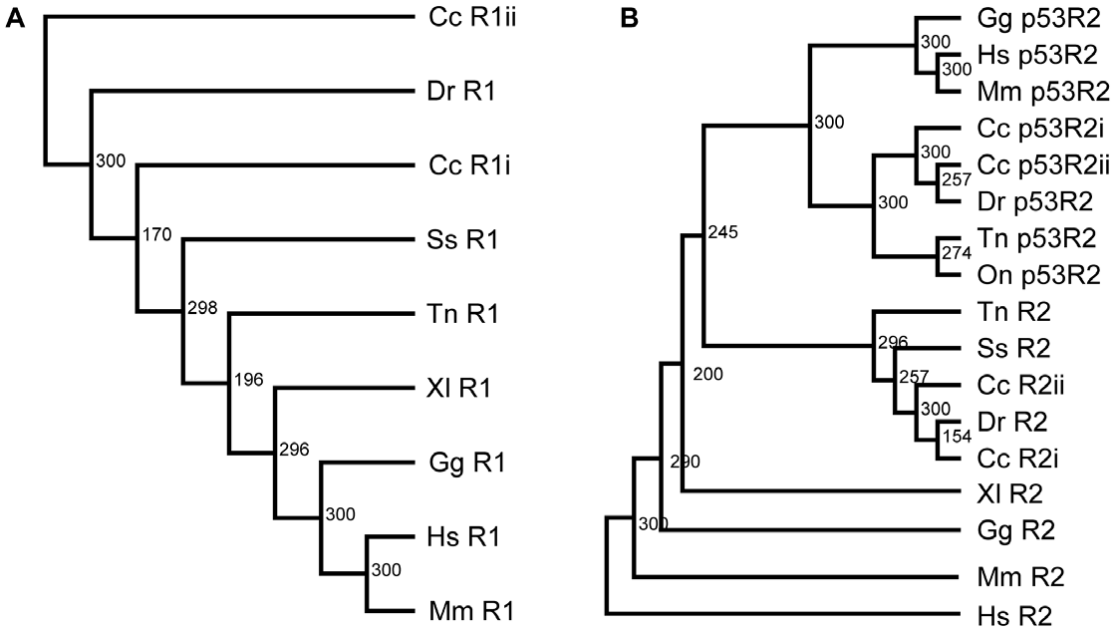
Phylogenetic trees of selected vertebrate RNR sequences. Panel (A) shows R1 subunits and panel (B) shows R2 and p53R2 subunits. Cc = *Carassius carassius* (crucian carp); Dr = *Danio rerio* (zebrafish); Ss = *Salmo salar* (Atlantic salmon); Tn = *Tetraodon nigroviridis* (green spotted pufferfish); On = *Oreochromis niloticus* (Nile tilapia); Xl = Xenopus laevis (African clawed frog); Gg = *Gallus gallus* (chicken); Mm = *Mus musculus* (mouse); Hs = *Homo sapiens* (human). The confidence scores of a bootstrap test of 300 replicates are indicated for branch nodes scoring over 150. The trees were made with PHYLIP.

Finally, the two RNR p53R2 paralogs, p53R2i (JQ679008) and p53R2ii (JQ679009) had CDS translating into 337 and 349 aa, respectively, and also showed a high degree of homology to mammalian RNR p53R2 sequences (see amino acid sequence alignment in Fig. S2). All functional sites listed above for R2 were also conserved in p53R2 variants. The phylogenetic trees made from the present sequences and other vertebrate RNR sequences found in GeneBank further supported that the RNR sequences in crucian carp were similar to RNRs in other vertebrates (Fig. 1). However, a KEN box is present at amino acid 24–36 in p53R2ii, which is not usually found in p53R2 subunits.

**Figure 2:**
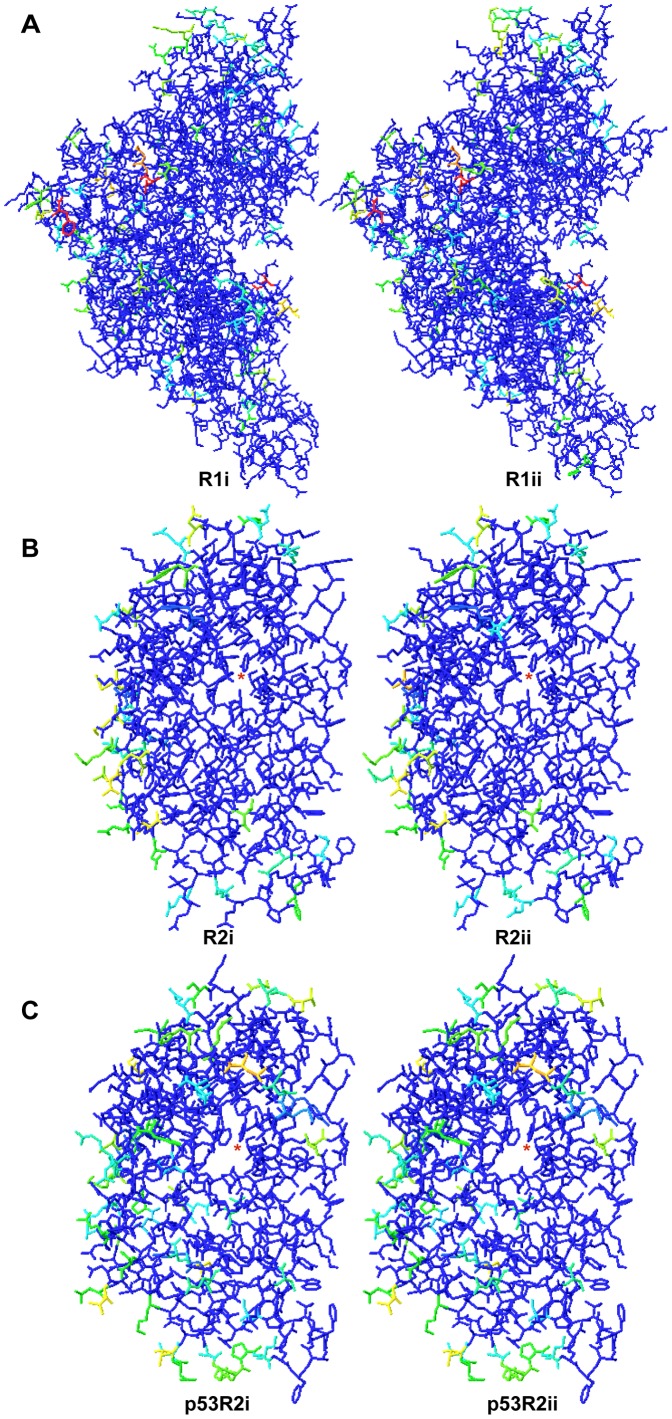
Homology modelling of crucian carp RNR subunits with mammalian RNR as templates. Panel (A) shows R1 subunits superimposed on human structure (PDB code 3HCN), panel (B) shows R2 subunits superimposed on mouse R2 structure (PDB code 1W68), and panel (C) shows p53R2 subunits superimposed on human p53R2 structure (PDB code 3HF1). Blue colour signifies strong homology in structure, green to yellow signifies intermediate homology, and red signifies low homology in structure. The tyrosyl radical site in each of the R2/p53R2 subunits is marked with a red star.

Homology modelling of crucian carp RNR with mammalian structures confirmed that crucian carp RNRs show strong homology to mammalian RNRs (Fig. 2). Most of the difference between crucian carp and mammalian RNRs were found in surface residues.

### Effect of anoxia on mRNA levels of cell division markers and RNR subunits

**Figure 3:**
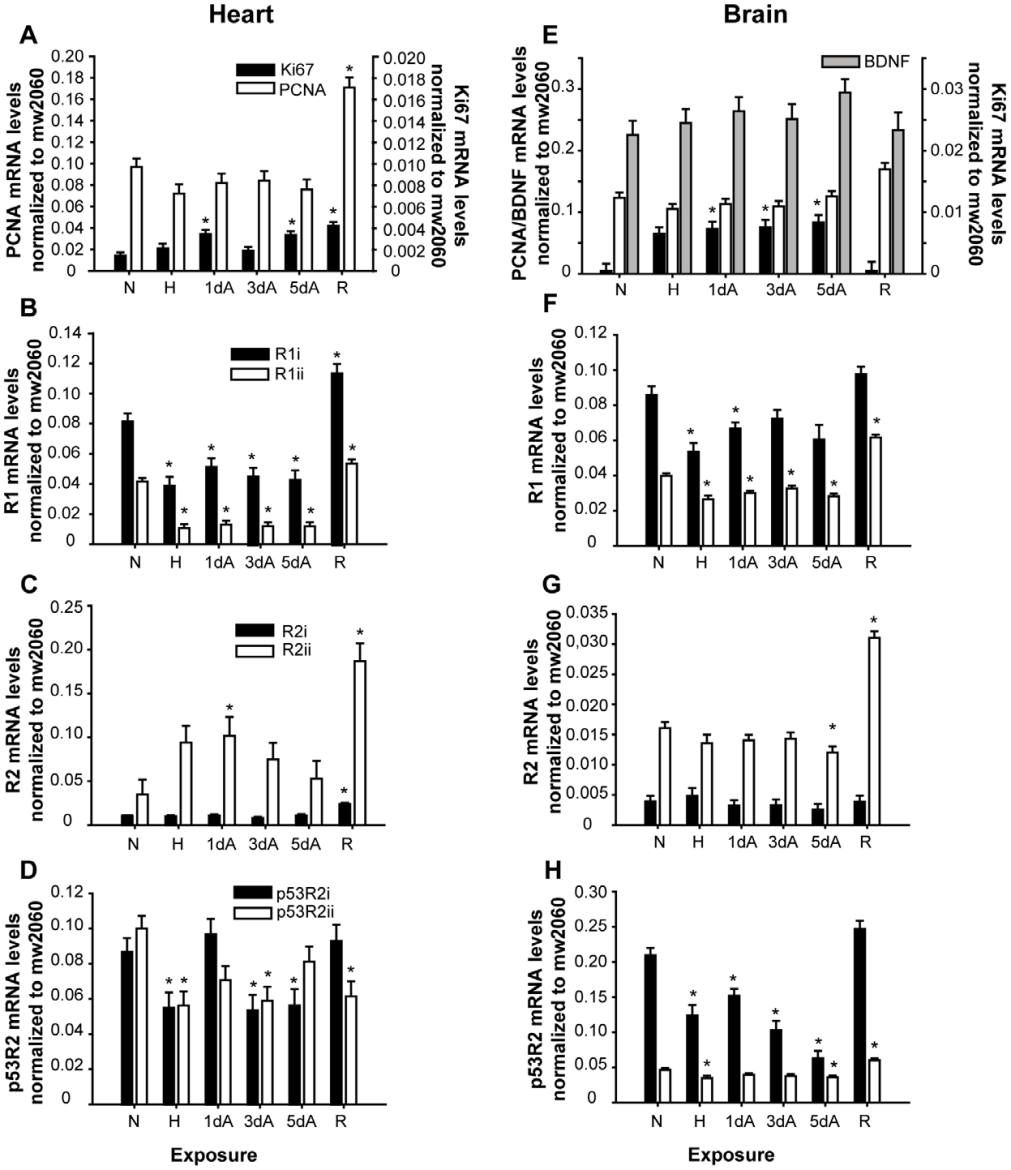
mRNA levels of cell division markers and RNR subunits at different oxygen levels. Panels (A–D) show heart mRNA levels and panels (E–H) show brain mRNA levels in normoxia (N), hypoxia (H), anoxia (A) and reoxygenation (R). All qPCR data are normalized to the external standard mw2060. d = duration of exposure (days). Significant differences from the normoxic group are indicated by asterisk (*P*<0.05). n = 6–10 in each group. Note that y-axis scales vary between panels.

Levels of mRNA for cell division markers (PCNA, Ki67, BDNF) and the RNRs (two paralogs for each of R1, R2 and p53R2) were measured with qPCR in brain and hearts from normoxic (control), hypoxic, anoxic and reoxygenated fish (Fig. 3). To avoid problems with unstable internal reference genes in anoxia, we normalized all qPCR data to an exogenous mRNA standard added during the homogenization (mw2060; see Methods).

Interestingly, the mRNA levels of the cell division markers were either maintained or increased during hypoxia and anoxia (Fig. 3A, E). Ki67 increased slightly after 1 and 5 days of anoxia in the heart, and increased about 10 fold in all anoxic groups in the brain. No changes compared to the control were seen in BDNF mRNA levels in hypoxia, anoxia or reoxygenation. At reoxygenation, the Ki67 mRNA level in brain returned to normoxic levels, while it continued to be higher than normoxia in the heart. The PCNA mRNA level in reoxygenated hearts was increased 2 fold compared to the normoxic value.

In the heart, the two paralogs of the large RNR subunit R1 showed lower mRNA levels in hypoxia and anoxia, and higher levels at reoxygenation (Fig. 3B). The same pattern could be seen in brain (Fig. 3F). The small subunit R2 mRNA levels were more or less maintained in anoxia in both tissues (Fig. 3C, G). However, the R2ii variant showed increased mRNA levels in the anoxic hearts, only significantly higher after 1 day of anoxia (Fig. 3C). It may be noted that the heart R2ii mRNA level was 2 fold higher than in brain under normoxic conditions, possibly indicating a higher basal cell division rate in heart compared to brain. The mRNA levels of R2 variants were generally increased during reoxygenation, with R2ii mRNA levels increasing 5 fold in heart. Finally, the general trend across tissues for p53R2 was lower mRNA levels in hypoxia/anoxia (Fig. 3D, H). The p53R2i mRNA returned to normoxic levels at reoxygenation in both tissues, while the other paralog p53R2ii decreased in heart and increased in brain in this group.

### EPR spectra of carp tyrosyl radicals

**Figure 4:**
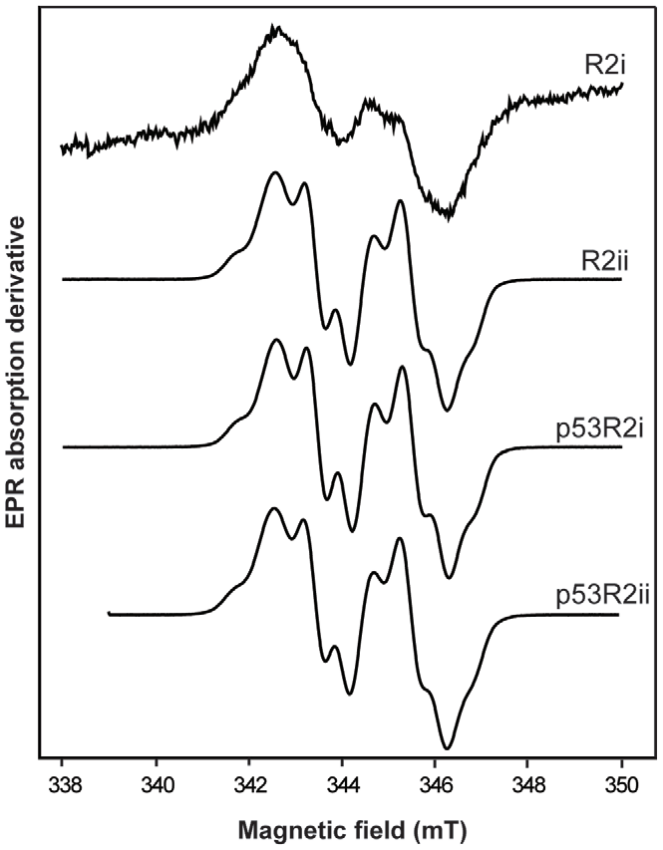
X-band EPR spectra of crucian carp tyrosyl radicals in RNR R2 and p53R2 variants. Spectra were obtained at temperature = 5 K, frequency = 9.67 GHz, modulation amplitude of 0.25 mT and microwave power = 0.4 mW. The crucian carp X-band spectra are very similar to mammalian spectra [46].

**Figure 5:**
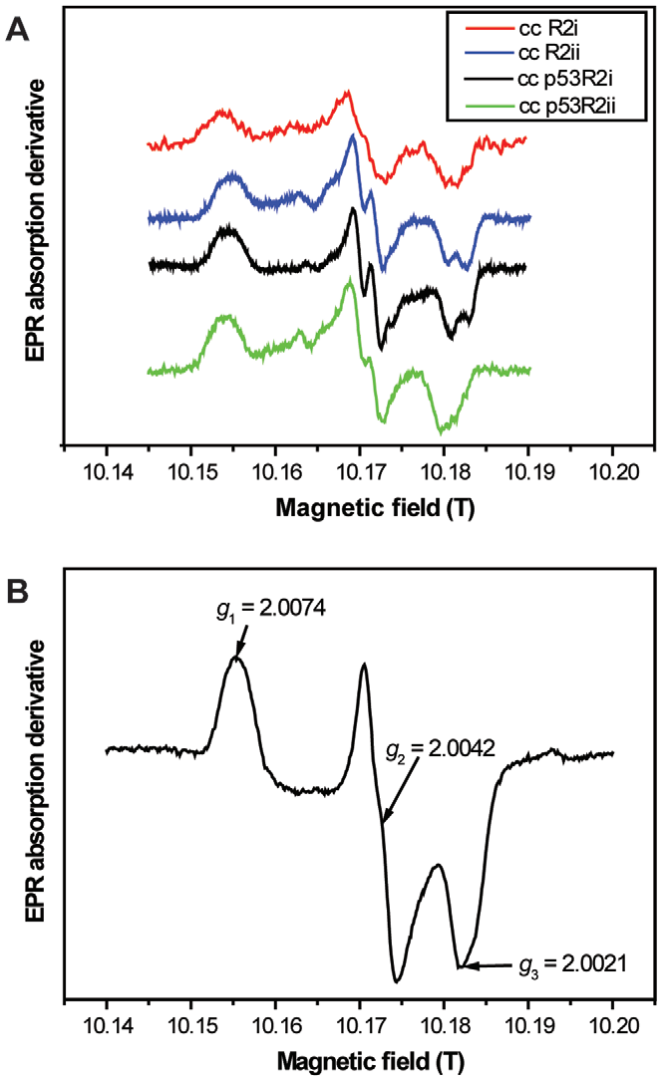
HF-EPR (285 GHz) spectra of crucian carp and human tyrosyl radicals in R2 and p53R2. Panel (A) shows all crucian carp R2 and p53R2 HF-EPR spectra, while panel (B) shows the human p53R2 spectrum with *g*-values indicated. Spectra were recorded at 15 K with a modulation amplitude of 0.4 mT, microwave power in the µW range and are an avarage of 4 scans.

The free tyrosyl radical from apo/metal-free crucian carp R2 and p53R2 proteins, reconstituted with Fe^II^ and O_2_, was studied at two EPR microwave frequencies, 9.6 GHz (X-band) and 285 GHz (HF-EPR). The observed first-derivative EPR spectra are shown in Figure 4 and 5, respectively. The X-band EPR envelope of R2ii, p53R2i and p53R2ii (Fig. 4) were similar to the spectra observed in mammalian R2 and p53R2 reported earlier [49], [50]. Limitations in the effective expression/purification of the R2i form led to small amounts of protein available for EPR measurements, and the protein concentration in this EPR sample was about one third compared to the other samples. Consequently, the observed EPR envelopes for the tyrosyl radical in the R2i protein has higher noise to signal ratios (Fig. 4A) than usually observed in RNR R2 tyrosyl radical spectra.

The tyrosyl radical in crucian carp RNR R2ii and p53R2ii was tested for stability as judged from the EPR signal. In p53R2ii it was very stable, both in anoxia and normoxia. After incubation for 24 h in anoxia at 0°C, there was no detectable decrease in the EPR signal from p53R2ii (n = 2). Further incubation at room temperature for 1 h in normoxic atmosphere did not result in any decrease in the EPR signal (n = 2). Incubation at room temperature for 1 h in normoxia alone gave the same result: no decrease in signal (n = 2). The radical in crucian carp RNR R2ii was somewhat less stable in anoxia, but still only a small decrease was seen in the EPR signal from the samples incubated in anoxia for 24 h at 0°C (a 31% decrease, n = 2). Further incubation at room temperature for 1 h in normoxia did not result in any additional decrease in EPR signal (n = 2).

The anisotropic *g*-tensor components are usually poorly resolved under X-band microwave frequency. The HF-EPR spectra (Fig. 5), on the other hand, show nicely resolved *g*-tensor anisotropy (Table 3). The *g*_1_-values for both crucian carp R2 paralogs were around 2.0073, which are lower than all other *g*-values identified in native R2 or p53R2 subunits.

**Table 3:** g-values for the tyrosyl radical in class Ia RNR.

	*g*_1_	*g*_2_	*g*_3_	Ref.
Crucian carp R2i	2.0073	2.0042	2.0022	Present study
Crucian carp R2ii	2.0073	2.0041	2.0022	Present study
Crucian carp p53R2i	2.0074	2.0042	2.0022	Present study
Crucian carp p53R2ii	2.0074	2.0042	2.0022	Present study
Mouse R2	2.0076	2.0043	2.0022	[51], [81]
*E. coli* R2	2.0090	2.0044	2.0021	[85], [86]
Epstein-Barr virus R2	2.0080	2.0043	2.0021	[44]
Herpes simplex virus type I R2	2.0076	2.0043	2.0022	[51], [81]
Human p53R2	2.0074	2.0042	2.0021	Present study
Mouse hp53R2	2.0078	2.0043	2.0022	Present study

The X-band and 285 GHz EPR spectra of the tyrosyl radical from crucian carp RNR R2ii, p53R2i and p53R2ii and human/mouse p53R2 (Fig. 5 and Table 3), can essentially be simulated using previously published mouse R2 spin-Hamiltonian parameters, since all these spectra feature very similar resonance signatures and hyperfine (A_H_) terms as those derived in mouse R2 ([51]; e.g. *g* = 2.0076, 2.0043, and 2.0022 for the components of the *g*-tensor; and hyperfine coupling constants (units in Gauss; G) A_β1_ = 21.4, 19.0, and 21.5; A_β2_ = 9.5, 2.5, and 5.7; A_3,5H1_ = −9.1, −4.4, and −6.6; A_3,5H2_ = −7.3, −4.8, and −5.8; line width = 4.5, 3.5, and 4.4 G), as all these spectra are almost identical to mouse R2 X-band spectra.

### The Fe^II^Fe^III^ form of carp R2 and p53R2 are similar to mammalian R2 and p53R2

The tyrosyl radicals in R2 proteins can be reduced to tyrosine, leaving a di-ferric Fe^III^Fe^III^ form with known 3D structure. However, this form does not display any detectable X-band EPR signal, as the two high spin μ-oxo-bridged ferric ions (S = 5/2) are anti-ferromagnetically coupled, generating a diamagnetic (S = 0) ground state spin-configuration [8], [52]. In mouse and herpes simplex R2 can, with mild reducing conditions, generate mixed-valent Fe^II^Fe^III^ forms [45], [53]. In the mixed-valent form, one iron is ferrous Fe^II^ high spin (S = 2) and the other iron remain ferric high spin (S = 5/2). The two irons are anti-ferromagnetically coupled resulting in a S = 1/2 ground state with an EPR signal. This signal has normally all three *g*-values falling below *g* = 2.0. Such fingerprints have been used as an indication of the presence of an oxy/hydroxy bridge between two irons [45], [52]. Many proteins in the iron-oxygen protein class (e.g. methane monooxygenase hydroxylase and purple acid phosphatases) can have this type of EPR active form [45], [52]. Mouse and herpes simplex virus type 1 have been reported to exhibit this specific EPR signal, while other class I R2 proteins like Epstein Barr virus R2 do not show such EPR feature, hence seem incapable to form mixed valent states for the iron-oxygen cluster.

**Figure 6:**
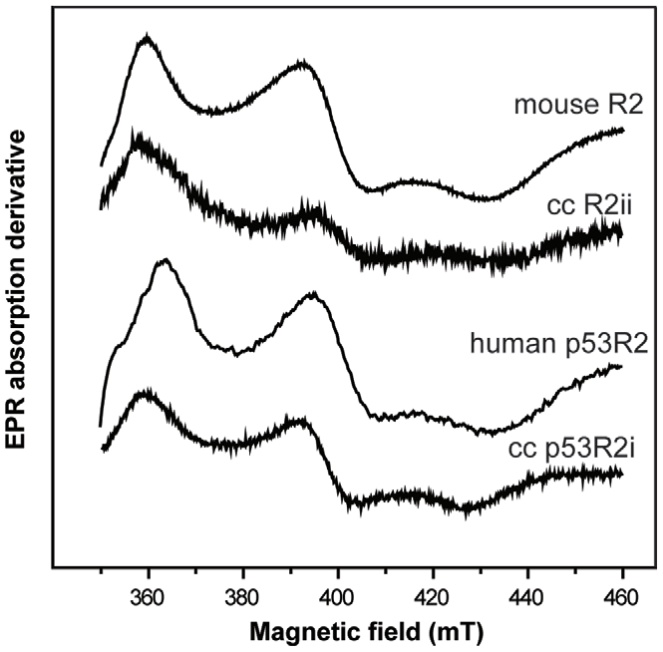
Mixed-valent EPR spectra of crucian carp R2ii and p53R2i compared to mammalian homologs.

Fig. 6 shows the EPR spectra of the Fe^II^Fe^III^ form of mouse R2 [similar to spectra reported in reference 45], human p53R2, crucian carp R2i, and crucian carp p53R2i. The four EPR spectra show similar signals. However, the EPR signals from crucian carp are not as broad as the two mammalian EPR signals. Mouse p53R2 has the broadest EPR signal (not shown), with *g*-values at 1.91, 1.72, and 1.59 (Table 4). The crucian carp R2s and p53R2s have resolved *g*-values (Table 4), while the *g*_3_-value in herpes simplex virus type 1 R2 is not so well resolved. Human and mouse p53R2s have similar yields of the mixed valence form as mouse R2, close to half of total iron can be obtained as an EPR active mixed valence Fe^II^Fe^III^ species. The crucian carp p53R2s are not formed to the same yield as mouse R2, but their behavior are similar to herpes simplex virus type 1 R2, where about one third of total iron can be obtained as an EPR active mixed valence Fe^II^Fe^III^ species under similar reducing conditions. For crucian carp R2s, about 7±3% of total iron can be observed in the mixed valence form.

**Table 4:** g-values for the mixed-valent form of RNR.

	*g*_1_	*g*_2_	*g*_3_	Ref.
Crucian carp R2ii	1.92	1.73	1.61	Present study
Crucian carp p53R2ii	1.91	1.72	1.61	Present study
Mouse R2	1.92	1.73	1.60	[45]
Mouse p53R2	1.91	1.72	1.59	Present study
Human p53R2	1.90	1.72	1.60	Present study
Herpes simplex virus type 1 R2	1.93	1.75	1.63	[45]

## Discussion

All vertebrates encode class Ia RNR and are therefore dependent on oxygen for dNTP production and consequently cell division. Because previous studies of the crucian carp indicate that it has cell division in anoxia [29], we set out to characterize all RNR variants in crucian carp. We wanted to find out if they were classical vertebrate class Ia RNR, or if their sequence or structure could indicate that they could function at very low oxygen levels or even without oxygen, enabling cell proliferation in anoxia.

### Crucian carp RNR subunits

Zebrafish and mammals have one gene coding for the RNR R1 subunit, one gene for the R2 subunits and one for the p53R2 subunit [54], [55]. We here present the full-length mRNA sequence of two variants for each RNR subunits present in zebrafish and mammals. Due to a large-scale duplication of DNA in the teleost ancestor, fish have potentially twice as many genes as tetrapods [56], [57]. However, gene loss or silencing of one paralog after genome duplication is common, resulting in just one of the paralogs being retained [58]. This seems to have happened with the RNR paralogs in zebrafish.

The *Cyprinus* (common carp) and *Carassius* (crucian carp and goldfish) lineage has experienced an additional genome duplication [32]–[34], leading to potentially twice as many gene variants as in zebrafish. For the RNR genes, both paralogs of each subunit have been retained in the crucian carp (Fig. 1). Furthermore, their mRNA levels show that both paralogs of each variant are expressed and even show differential mRNA expression in response to varying oxygen levels, indicating that both paralogs of each subunit are functional (Fig. 3).

With two copies of each gene, one copy can gain a new function, without the organism losing the old function. Thus, it is thought that genome duplication is a major promoter of evolutionary inventions [58]–[60]. Sequence analysis and phylogenetic analysis indicate that no mutations have occurred in any of the important sites for radical formation, iron binding or radical transport in crucian carp RNR subunits. However, we found one mutation that lead to the presence of a KEN box in one of the p53R2 paralogs, p53R2ii. This box is not found in p53R2 subunits in other species, but is present in R2 subunits in all species, also in crucian carp R2i and R2ii. The KEN box is recognized by the anaphase promoting complex (APC) and is thought to be responsible for the degradation of R2 protein in late mitosis [61]. The presence of this box in crucian carp p53R2ii could indicate that it has overlapping functions with R2, i.e. it may provide nucleotides for cell division.

All the RNR variants cluster nicely together with their mammalian counterparts in the phylogenetic trees (Fig. 1). Furthermore, homology analysis with structures made from the crucian carp sequences based on mammalian RNR structures indicate a similar structure to mammalian RNRs (Fig. 2). Thus, it seems that the crucian carp RNR sequences found in this study code for typical vertebrate class Ia RNRs, dependent on oxygen for radical formation.

### RNR and cell division marker mRNA levels in anoxia and reoxygenation

Vertebrate cells normally go into cell cycle arrest in hypoxia [62], [63]. In contrast to this, we found that the cell division markers in both brain and heart showed maintained or even increased mRNA levels (Fig. 3). We used three different markers for cell division, PCNA, Ki67 and BDNF. PCNA is a cofactor of the DNA polymerase and is essential for DNA replication [64] and its protein and mRNA level is commonly used to mark dividing cells, also in fish [65]. BDNF has several functions in brain, and its expression is linked to neurogenesis and survival of new neurons [66]. While the mRNA levels of PCNA and BDNF were relatively constant during the anoxic period, the mRNA level of Ki67 increased in anoxia in the brain and in some anoxic groups in the heart. Ki67 is widely used as a marker of cell division, because of its presence in proliferating cells [67]. However, recently it has been shown that it also has other functions, as in chromatin remodelling and ribosomal RNA synthesis in non-dividing cells [68]. Taken together, the present results suggest that cell cycle progression does not stop in crucian carp heart or brain during anoxia. These results support the previous finding of maintained levels of cell division in intestine and liver after 7 days of anoxia, measured histologically by BrdU-incorporation and PCNA staining [29]. In contrast to adult mammals, adult teleosts have substantial cell division in both brain and heart as they continue to grow throughout life [69], [70]. It has been shown that the crucian carp maintains full cardiac activity [71] and partial brain activity in anoxia [72], [73], which may indicate that it needs cell division during long-term anoxia to maintain tissue integrity.

Also for the RNR R2 subunits, we found maintained or even transiently increased mRNA levels in anoxia, and increased levels at reoxygenation. As mentioned in the introduction, it is normally the presence of the R2 protein that regulates the activity of the RNR enzyme in the cell cycle, as it is degraded in mitosis [20]–[22]. It has been shown that the crucian carp maintains protein synthesis in brain during anoxia, and protein synthesis continues also in the anoxic heart, albeit at a reduced level [74]. However, without oxygen, any R2 proteins synthesized in anoxia can probably not generate a radical, and may remain as apoproteins until oxygen reappears. The maintained levels of R2 mRNA could function as a preparation for reoxygenation, enabling rapid production of dNTPs when oxygen reappears.

mRNA levels of both R1 paralogs decreased in anoxia in both brain and heart. However, the mRNA level stabilized at 30–50% of normoxic levels, and did not show any further decrease after the first anoxic day. Interestingly, the half-life of RNR R1 protein in mammals is long, and it can thus be present in the cells even when its mRNA levels are low [23]. mRNA levels of R2 and R1 subunits generally increased at reoxygenation in heart, correlating with the increased mRNA levels of the cell division markers, probably reflecting a need for replacement of cells after anoxia. Also in brain, R2ii and R1ii mRNA levels increased at reoxygenation, possibly because the brain may need some replacement of cells after anoxia.

The p53R2 subunit has been proposed to be necessary for DNA repair, to provide the R1 subunit with a radical outside S-phase, and is expressed in all phases of the cell cycle [19], [25], [26]. In the crucian carp brain and heart, mRNA levels of both p53R2 variants showed generally lower levels in hypoxia and anoxia, and only the p53R2ii variant increased at reoxygenation in brain. This suggests that it is not a major need for DNA repair after reoxygenation in crucian carp heart or brain. However, it is possible that damaged DNA is repaired very soon after anoxia, and an increased expression of p53R2 would not be seen after five days of reoxygenation.

### EPR spectroscopy of the tyrosyl radical environment and di-iron-oxygen cluster

EPR spectroscopy is used to detect magnetic moments of unpaired electrons in radicals and metal ions [75], and has been widely used for studying the radical site in RNR R2 subunits [76], [77]. The tyrosyl radical in crucian carp RNR R2 and p53R2 gave very similar EPR spectra as mammalian variants (Fig. 4) supporting the results from the sequence analyses showing that crucian carp RNR is a typical class I RNR. Furthermore, the spectra and the *g*-values of the mixed-valent state of crucian carp are very similar to the those found in mouse R2 and human p53R2 (Fig. 6 and Table 4), which indicates that the structure of the di-iron-oxygen cluster of crucian carp R2/p53R2 bears a close resemblance to the structure of the di-iron-oxygen cluster known in mammalian R2/p53R2.

The HF-EPR measurements allowed to resolve the *g*-tensor values associated to the tyrosyl radical. The *g*_1_ value, in particular, has been found to vary considerably in presence of hydrogen bonding interactions between the phenoxyl oxygen of the tyrosyl radical and, for example, a water molecule close to the radical site [51], [77]–[79]. Large *g*_1_ values such as *g*_1_ = 2.0090 for *E. coli* R2 (Table 3) are consistent with absence of a hydrogen bond [79], [80]. Hydrogen bonding interactions of different strength can give lower *g*_1_-values, exemplified by *g*_1_ = 2.0076 as found in mouse R2, which has a D_2_O exchangeable proton (probably from water) near the tyrosyl oxygen (-O…H, 1.89 Å) [51], [81]. Interestingly, we found that both crucian carp R2 paralogs had a *g*_1_ value of 2.0073, which is the lowest *g*_1_ value measured in a native RNR R2, which may indicate that the presence of strong hydrogen bonding to the tyrosyl radical in crucian carp (Table 3). However, as recently discussed for EBV R2, which exhibits an intermediate *g*_1_ value of 2.0080 falling between those of *E. coli* and mouse R2, such a shift in *g*_1_ should still be taken with caution as a direct evidence of hydrogen bonding interactions [44].

In *E. coli* RNR R2, the di-iron center is protected inside the protein, and the tyrosyl radical is stable for weeks [82]. As mentioned, the EPR spectra of the tyrosyl radical in *E. coli* show a high g_1_ value, and it has no hydrogen bond. In contrast, mammalian R2 di-iron-oxygen cluster is accessible to the solvent through an open aqueous channel [83], [84], and the half-life of the radical in mammalian RNR has been found to be in the range of 10–60 min at 37°C [16], [18]. Strong hydrogen bonds, indicated by the low g_1_ value in mammalian R2 as described above, has been suggested to stabilize the tyrosyl radical [81]. Homology modelling based on sequence alignment suggests that the crucian carp R2 paralogs also have aqueous channels, and the finding of a low *g*_1_-value of both paralogs of crucian carp R2 can indicate the presence of a hydrogen bond, which may serve as protection from the aqueous pore, as in mammals.

Interestingly, the crucian carp RNR R2 subunits, and especially the p53R2ii seemed to be very stable. After 24 h of anoxic incubation at 0°C, the EPR signal from the tyrosyl radical of p53R2ii was not decreased compared to a normoxic control (immediately frozen after reconstitution). The EPR signal from the tyrosyl radical in R2ii was only decreased by about 30% in the same experiment. Further incubation of the samples for 1 h in normoxia at room temperature did not lead to any decrease in the signals from any of the samples. In a similar experiment with mouse RNR R2, 10 minutes of anoxic incubation at 37°C was enough to decrease the EPR signal with about 40% [16]. In another experiment mouse R2 has been shown to loose about 50% of its activity after 20 min incubation in anoxia at 37°C [18]. Even if the temperature was different in these experiments compared to the present study, it indicates that crucian carp p53R2ii is very stable at a temperature relevant for anoxic overwintering. Interestingly, as discussed above, this variant contains a KEN box, which is normally only found in R2 subunits, which could indicate that p53R2ii can possibly function in supplying nucleotides for DNA synthesis during cell division.

### Concluding remarks

In the present study, we show that crucian carp express two paralogs of each of the three known vertebrate RNR subunits. This, together with the great similarity that these variants show to those of other vertebrates, suggests that the functions of all paralogs have been maintained after the gene duplication event of the *Carassius* lineage. The mRNA levels of the R1 paralogs and p53 paralogs showed an initial decrease followed by stabilization during anoxia in brain and heart. In contrast, the mRNA level of R2 subunits did not decrease in hypoxia or anoxia, which could probably be an advantage for rapid production of nucleotides when oxygen reappears in the spring.

Furthermore, we showed that the mRNA levels of several cell division markers were maintained in anoxia, possibly pointing at continued mitotic activity in brain and hearts of anoxic crucian carp. Finally, we found that the tyrosyl radicals in RNR are very stable in anoxia at low temperature, which may enable continued RNR activity and therefore cell division during anoxia.

## Supporting Information

Figure S1 **Amino acid sequence alignments of fish and mammalian RNR R1 subunits.** RNR R1 sequences from human (Hs), mouse (Mm), zebrafish (Dr) and crucian carp (Cc) are included. Shading of the amino acids indicates homology between the sequences.
(TIF)

Figure S2 **Amino acid sequence alignments of fish and mammalian RNR R2 and p53R2 subunits.** RNR R2 and p53R2 sequences from human (Hs), mouse (Mm), zebrafish (Dr) and crucian carp (Cc) are included. Shading of the amino acids indicates homology between the sequences.
(TIF)
