# Supplementary figures and images for: Studies of Ribonucleotide Reductase in Crucian Carp—An Oxygen Dependent Enzyme in an Anoxia Tolerant Vertebrate
Source: PLoS One. 2012 Aug 14;7(8):e42784. doi: 10.1371/journal.pone.0042784 (PMC3419237; doi:10.1371/journal.pone.0042784)

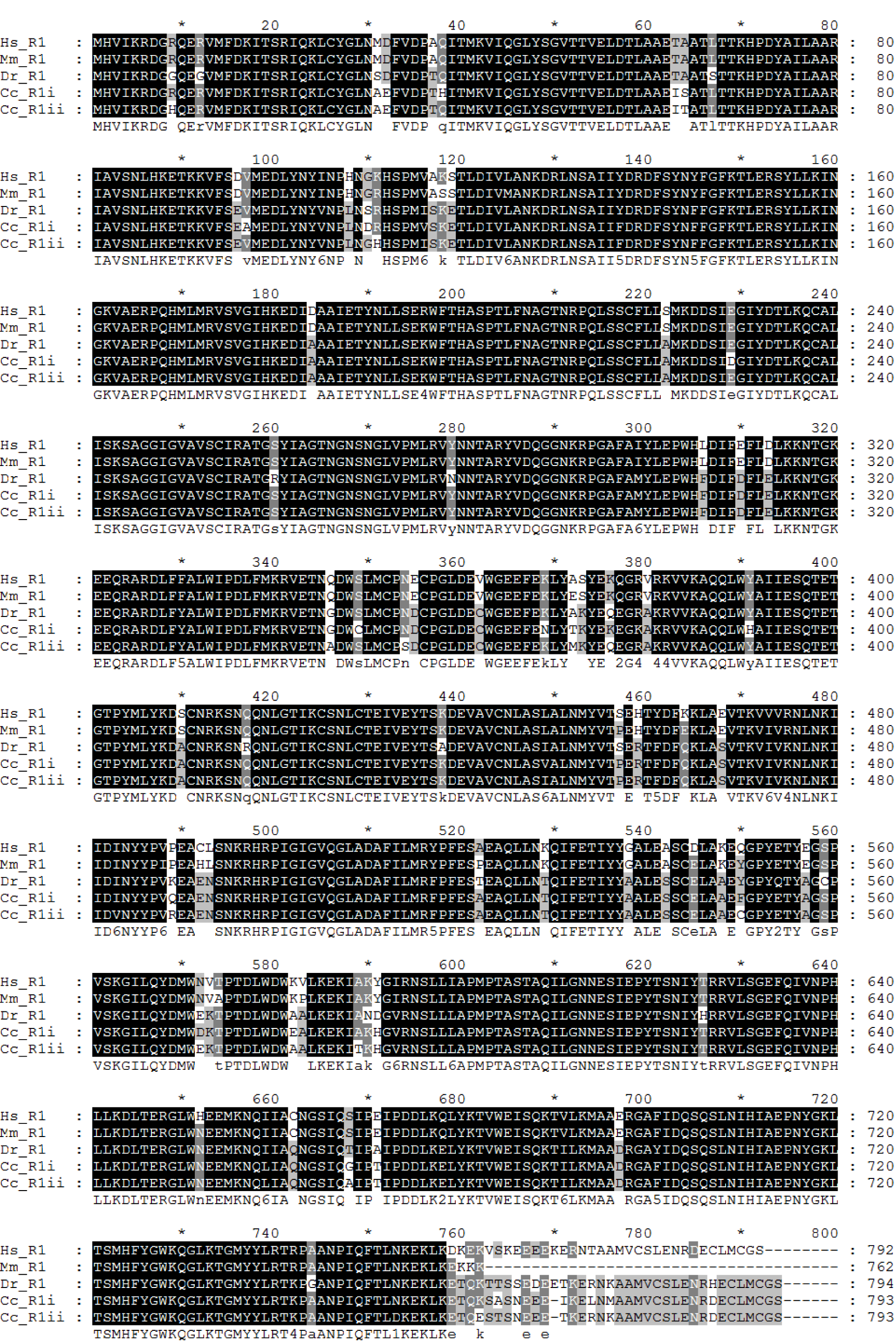

Supplement: Figure S1 — Amino acid sequence alignments of fish and mammalian RNR R1 subunits. RNR R1 sequences from human (Hs), mouse (Mm), zebrafish (Dr) and crucian carp (Cc) are included. Shading of the amino acids indicates homology between the sequences. (TIF) [file pone.0042784.s001.tif]

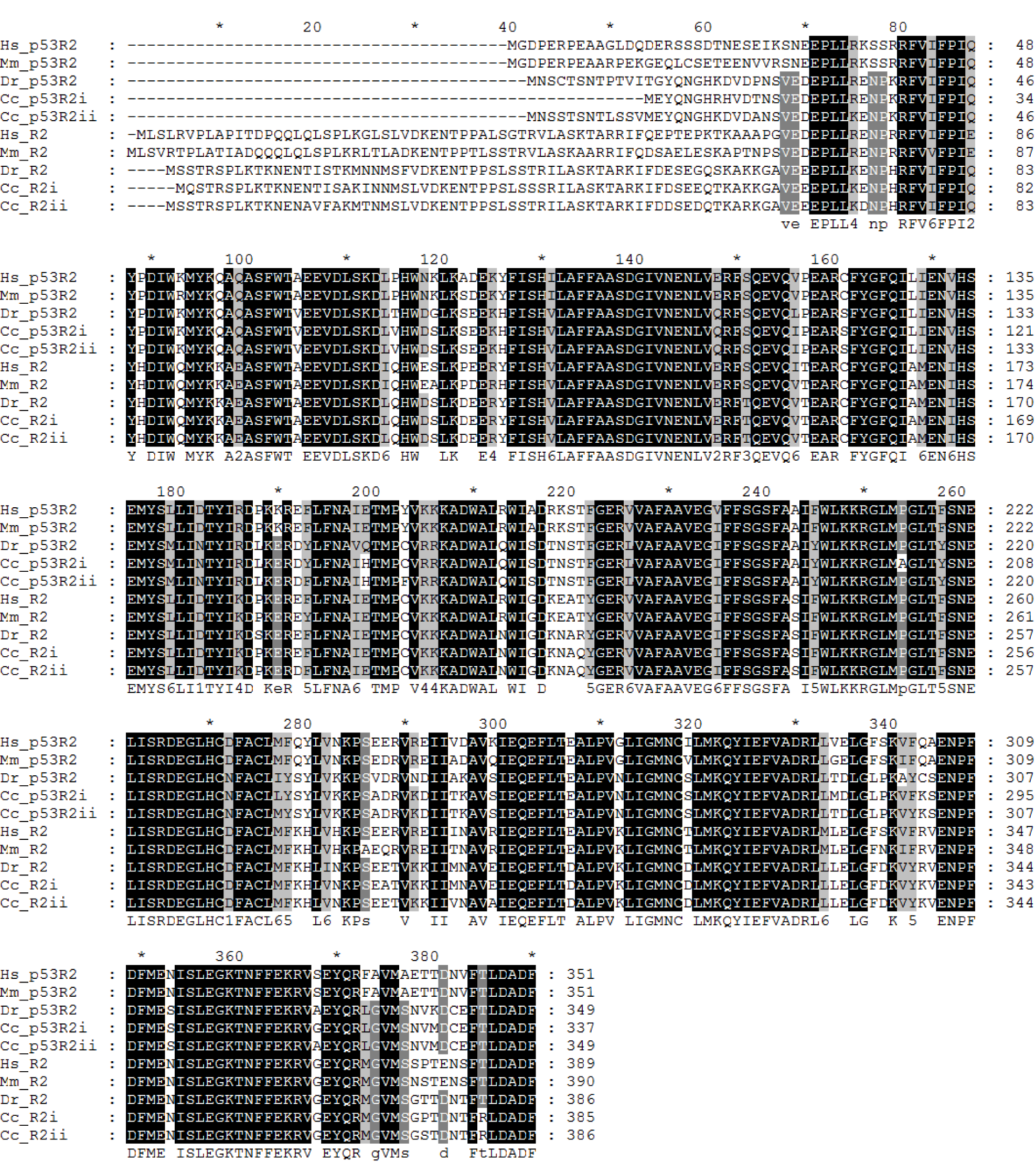

Supplement: Figure S2 — Amino acid sequence alignments of fish and mammalian RNR R2 and p53R2 subunits. RNR R2 and p53R2 sequences from human (Hs), mouse (Mm), zebrafish (Dr) and crucian carp (Cc) are included. Shading of the amino acids indicates homology between the sequences. (TIF) [file pone.0042784.s002.tif]
